# Relationships between Milk and Blood Biochemical Parameters and Metabolic Status in Dairy Cows during Lactation

**DOI:** 10.3390/metabo12080733

**Published:** 2022-08-09

**Authors:** Biljana Andjelić, Radojica Djoković, Marko Cincović, Snežana Bogosavljević-Bošković, Milun Petrović, Jelena Mladenović, Aleksandar Čukić

**Affiliations:** 1 Department of Animal Science, Faculty of Agriculture—Kruševac, University of Niš, Kosančićeva 4, 37000 Kruševac, Serbia; 2 Department of Animal Science, Faculty of Agronomy—Čačak, University of Kragujevac, Cara Dušana 34, 32000 Čačak, Serbia; 3 Department of Veterinary Medicine, Faculty of Agriculture, Novi Sad, University of Novi Sad, Trg Dositeja Obradovića 8, 21000 Novi Sad, Serbia; 4 Faculty of Agriculture, University of Priština, 38219 Lešak, Serbia

**Keywords:** cow, metabolic status, blood, milk, lactation

## Abstract

This study aimed to determine blood and milk metabolic parameters and their correlations for the purpose of evaluating metabolic status in dairy cows. Blood and milk samples were collected from 100 Holstein dairy cows during morning milking. The cows were allocated to four groups according to the production period, including cows in early (*n* = 18), full (*n* = 26), mid (*n* = 25) and late (*n* = 31) lactation. The value of non-esterified fatty acids (NEFA), β-hydroxybutyrate (BHB), glucose, triglycerides (TG), total cholesterol (TChol), total protein (TP), albumin, globulin, urea, total bilirubin (TBil), aspartate aminotransferase (AST), alanine aminotransferase (ALT), alkaline phosphatase (ALP), gamma-glutamyl transferase (GGT), and lactate dexydrogenase (LDH) in the blood were determined. The following milk parameters were measured: fat, protein, lactose, urea, AST, ALT, ALP, GGT, LDH and BHB. Blood serum NEFA, BHB, TBil, AST, ALT, ALP and LDH were higher in early lactation cows, whereas glucose, TP, globulin and urea levels were significantly lower in early lactation cows. Milk fat and lactose levels were lower in early lactation cows, whereas milk protein and the activities of AST, ALT, ALP and LDH in milk were highly greater in early lactation cows. Milk fat was positively correlated with glucose, TP and TG, and negatively correlated with BHB, NEFA, TBil, ALT, LDH and ALP levels in the blood. Enzyme activities in milk were positively correlated with those in blood and with blood NEFA, BHB and TBil levels, and negatively correlated with blood glucose, TChol and TG. A significant positive correlation existed between blood and milk BHB values. Many correlations showed the same slope during all lactation periods. In conclusion, similar changes in blood and milk metabolite concentration during lactation and milk to blood correlations confirm that milk has great potential in predicting of blood metabolites and metabolic status of cows.

## 1. Introduction

Dairy cows are commonly affected by production diseases, i.e., diseases related to poor nutrition or management [1,2]. Metabolic profile testing is routinely used to reveal metabolic disorders in dairy cattle. During lactation, blood and milk biochemical parameters are usually checked to evaluate animal health and milk yield, especially when the herd is at high risk of developing metabolic diseases [3,4,5,6]. Major health disorders in high-yielding cows occur around parturition and during lactation. States of negative energy balance (fasting, parturition and lactation) cause excessive fat mobilization and accumulation in liver cells, causing ketogenesis and disturbance of liver physiology and morphology [7,8,9,10]. 

Clinical and laboratory monitoring during the transition period and early lactation play an important role in detecting subclinical nutritional and metabolic diseases. The main blood markers of lipid mobilization in dairy cows are BHB, the most important ketone body, and NEFA [11,12,13]. Lipid metabolites are important in pathogenesis of many metabolic disease and stress adaptation in cows [14]. NEFA are accumulated as TG in the liver, primarily due to the decreased hepatic synthesis of very low-density lipoproteins (VLDL). However, in liver lipidosis, endogenous synthesis decreases, leading to a reduction in blood glucose, TP, albumin, globulin, TChol, TG and urea concentrations. Moreover, as the excretory capacity of liver cells is reduced, the blood levels of some metabolites such as TBil, ammonia and bile acids are generally elevated [15,16]. Fatty liver and diffuse infiltration of hepatocytes are characterized by cell membrane damage, hepatocyte destruction, and the release of cytoplasmic enzymes, whose blood activities are considerably increased. Therefore, blood serum ALT, aspartate aminotransferase (AST), ALP, LDH and GGT activities are useful indicators of postpartum liver function [8,17,18]. As there is little information about changes in ALT, AST, LDH, GGT and ALP activities in milk, practical attention has been focused on the assessment of enzyme activities in milk, with many enzymes being proposed and listed as reliable markers for early diagnosis of subclinical disease [19,20,21]. The activities of these enzymes in milk and blood sera of cows were evaluated and exhibited close relationships, as shown by the results of correlation analysis and regression models [22,23]. 

Milk parameters originate from blood and food components, and elucidating relationships among these parameters individually in food, blood and milk helps to understand the health and production status of animals [21,22,23,24,25]. Milk may be a preferred matrix whenever possible because it is non-invasive and easy to collect, and it is often used to identify ketosis and other production disease [26,27]. Variations in milk fat and milk BHB levels indicate lipomobilization and ketogenesis [28,29]. Milk, the outcome of various biochemical activities in mammary secretory cells, contains fat, protein, lactose, enzymes, vitamins and various minerals. Its composition is dependent on various factors, i.e., stage of lactation, lactation number, breed, feeding pattern, environmental conditions and udder health [30].

However, modern management practices involve more frequent or even daily monitoring of the health status of high-yielding dairy cows to control milk yield and quality and address potential health problems. This study aimed to determine blood and milk metabolic parameters and their relationships for the purpose of evaluating the metabolic status of dairy cows for early diagnosis of subclinical metabolic disease at different stages of lactation.

## 2. Materials and Methods

### 2.1. Animals and Study Design

A total of 100 dairy cows were randomly selected from the same Holstein herd containing almost 1000 cows (Vrbas, Vojvodina, Serbia). Clinically healthy cows were allocated to four experimental groups: Group 1—early lactation cows (*n* = 18), from 1 to 49 days of lactation; Group 2—full lactation cows (*n* = 26), from 50 to 109 days of lactation; Group 3—mid-lactation cows (*n* = 25), between 110 and 209 days of lactation; and Group 4—late lactation cows (*n* = 31), from 210 to 305 and more days of lactation. The cows were high-yielding, aged 4 years on average (with an average of 2.7 lactations), with a preceding lactation of about 8500L (average weekly yield was 26.5 L/cow/day). The average body condition score (BCS) was 3.36 ± 0.55 for all experimental cows. The experimental cows were housed in free-stall barns. Diet and housing conditions were adapted to the purposes of the experiment, with diet tailored to the cows’ energy requirements during different periods of lactation using National Research Council (NRC) standards [31]. 

On the farm, the experimental dairy cows were fed a total mixed ration (TMR) twice daily and had ad libitum access to water. The ration for early lactation cows contained: dry matter (DM) 21.5 kg; net energy of lactation 153.2 MJ; crude protein (CP) 18.3% DM; rumen undegradable protein 39.69% CP; fat 4.92% DM; fiber 17.2% DM; acid detergent fiber (ADF) 22.6% DM, and neutral detergent fiber (NDF) 37.16% DM. Generally, diets were designed to provide the consumption of about 23 kg DM, i.e., 43 kg of feed. At different stages of lactation, diets were balanced to increase the percentage of corn silage from 30% in the early lactation period to 50% in the dry period, whereas the grain proportion was reduced from 5–8% to 3–4% in respective periods. Balancing did not change the overall average chemical composition of the diet. 

### 2.2. Blood Analysis

Blood samples were taken 4 to 6 h after milking and feeding from the coccygeal vein into evacuated serum separator tubes. After clotting for 3 h at 4 °C and centrifugation (1500 G, 10 min), blood sera were analyzed for the following biochemical parameters: glucose, non-esterified fatty acids (NEFA), β-hydroxybutyrate (BHB), total cholesterol (TChol), triglycerides (TG), total bilirubin (TBil), total protein (TP), urea, albumin, globulin, aspartate aminotransferase (AST), alanine aminotransferase (ALT), alkaline phosphatase (ALP), gamma-glutamyl transferase (GGT) and lactate dexydrogenase (LDH), which were determined by colorimetric kits (Biosystem, Spain and Randox, Carlisle, UK) and a Chemray spectrophotometer (Rayto, Shenzhen, China). All analyses were performed at the Laboratory of Pathophysiology, Department of Veterinary Medicine, University of Novi Sad. 

### 2.3. Milk Analysis

Milk samples were collected during morning milking into tubes with and without additives on the same day blood was sampled. The chemical composition of milk was determined at the Central Laboratory for Milk Quality Control at Agriculture faculty of Novi Sad. Milk samples were analyzed by a FOSS milk analyzer, and their chemical composition was assessed by a MILKOSCANFT analyzer (Milko-Scan 133 B, Foss Electric, Denmark) using Fourier-transform infrared spectroscopy. Milk fat, protein and lactose contents were determined. Before analysis, samples were heated in a water bath at 40 + 2 °C. After homogenization, about 5 mL of milk was taken by the apparatus. Upon serum separation, milk was subjected to biochemical tests for the determination of the enzymes (AST, ALT, ALP, GGT, LDH), urea and BHB. Milk serum was separated after centrifugation at 10,000× *g* for 30 min and was transferred to new tubes for analysis. The biochemical reagents and apparatus used for milk serum analysis were the same as for blood serum.

### 2.4. Statistical Analysis

All blood and milk metabolic parameters were included in PCA analysis and unit variance scaling was applied to rows. Singular value decomposition with imputation was used to calculate principal components. The X and Y axes showed principal component 1 and principal component 2. Principal components analysis (PCA) was used to obtain a graphical representation of the four groups of cows to estimate which stage of lactation was expected to show the largest deviation in the parameters tested. Then, the effect of lactation period on blood and milk biochemical parameters was examined by ANOVA analysis coupled with an LSD post hoc test. Associations between milk and blood biochemical parameters were determined by Pearson’s coefficient of correlation. Importantly, estimating lipolysis and ketogenesis in dairy cows through milk parameters allowed continuous monitoring of energy balance. Regression lines between blood NEFA and BHB values and the parameters with which they were significantly correlated at the entire lactation level were derived. Then, the homogeneity of slopes of the regression lines between NEFA and BHB levels and milk parameters was tested as a function of lactation period to determine which parameters can be used throughout lactation, and which milk parameters should be considered relative to lactation period for the estimation of lipolysis and ketogenesis, i.e., the energy balance (EB). SPSS statistics software (IBM, USA) was used.

## 3. Results

**Figure 1:**
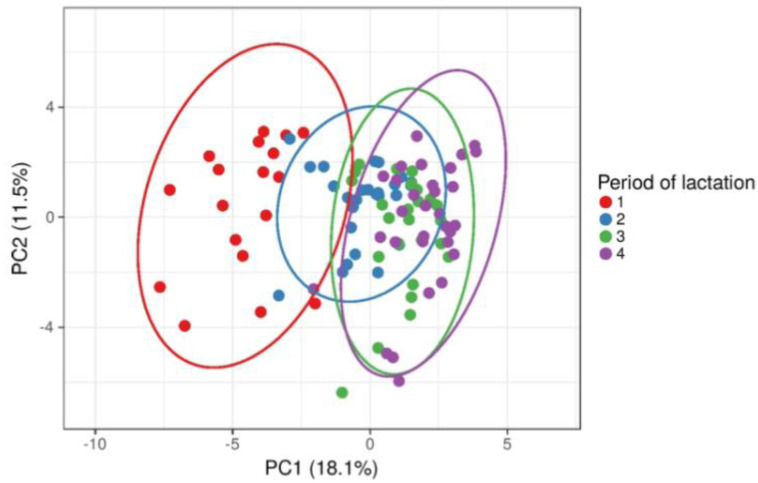
Principal components analysis (PCA) and clustering of cows based on lactation period (early—1, mid—2, full—3 and late—4) after including all blood and milk metabolic parameters.

The PCA score plots showed that cows from different period of lactation were clustered differently so that early lactation cows were clustered separately from cows in other three period of lactation (Figure 1). Considering that all metabolic parameters from blood and milk were included in the PCA analysis, the obtained result requires further examination of statistically significant differences between lactation periods for each metabolic parameter.

Blood biochemical parameters in cows during the four lactation periods are summarized in Table 1. Serum NEFA and BHB concentrations were highly significantly greater in early lactation cows than in cows during the other lactation periods (*p* < 0.001), whereas mean TG and TChol concentrations were significantly lower (*p* < 0.01) in early lactation cows. In addition, glucose, TP, globulin and urea levels were significantly lower in Group 1 (early lactation) cows (*p* < 0.01), which also exhibited a slight (but not significant) decrease in albumin concentration. By contrast, TBil and serum AST, ALT, ALP and LDH activities were significantly increased in early lactation cows compared with cows in the other periods of lactation (*p* < 0.01).

Table 2 shows significant changes in most milk metabolic parameters between the experimental groups of cows in this study. Milk fat levels were significantly lower (*p* < 0.001) in early lactation cows than in the other periods of lactation, whereas the mean values of milk protein were highly significantly greater (*p* < 0.001) in early and late lactation than in the other periods of lactation. Milk lactose levels were significantly lower (*p* < 0.001) in early lactation cows than during mid and late lactation. The activities of AST, ALT, ALP and LDH in milk were also significantly higher in early lactation (*p* < 0.01) than in the other stages of lactation. There was no significant difference (*p* > 0.05) in milk serum BHB and urea values between the experimental groups of cows. 

Table 3 shows the coefficients of correlation between blood and milk biochemical parameters calculated for all cows in this experiment. Milk fat was positively correlated (*p* < 0.05) with glucose, TP and TG, and negatively correlated (*p* < 0.05) with BHB, NEFA, TBil, ALT, LDH and ALP levels in the blood. There was a significantly positive correlation (*p* < 0.05) between milk protein and blood GGT. Enzyme activities in milk were positively correlated (*p* < 0.05) with those in blood (except for LDH) and with blood NEFA, BHB and TBil levels, and negatively correlated (*p* < 0.05) with blood glucose, TChol and TG. A significant positive correlation (*p* < 0.01) existed between blood and milk BHB values. 

**Figure 2:**
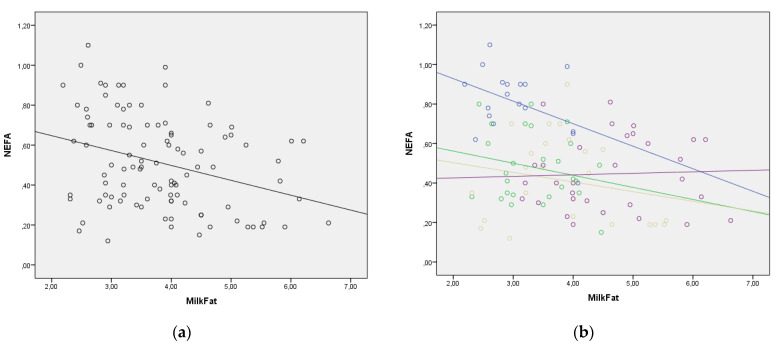

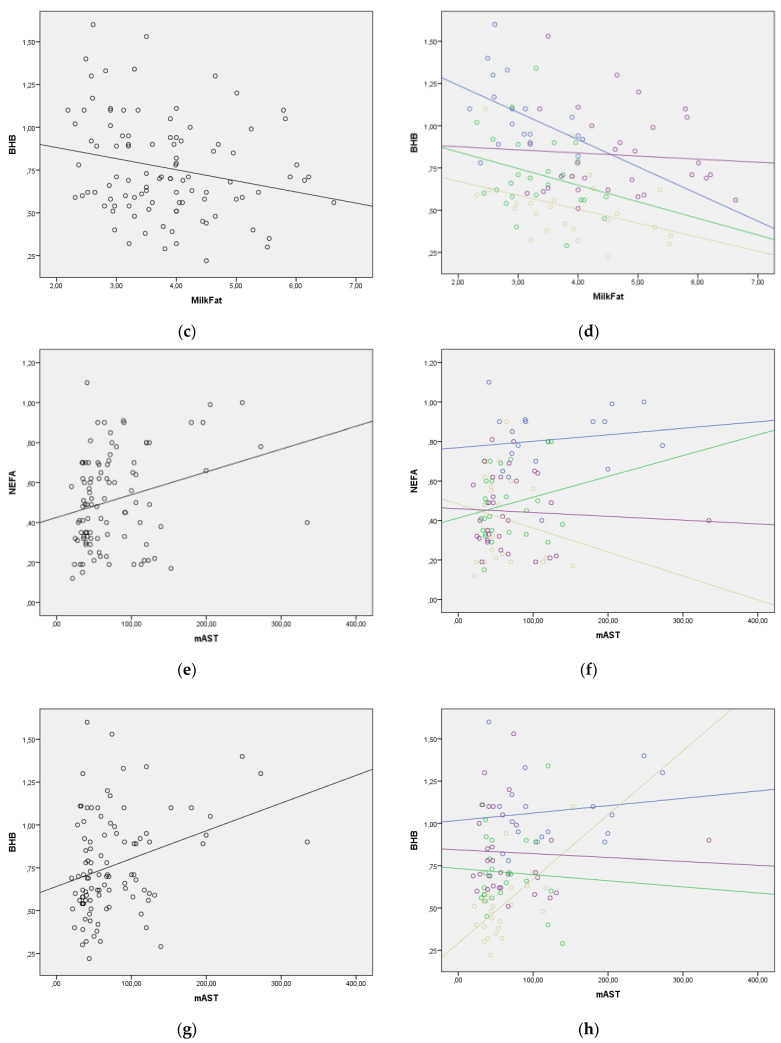

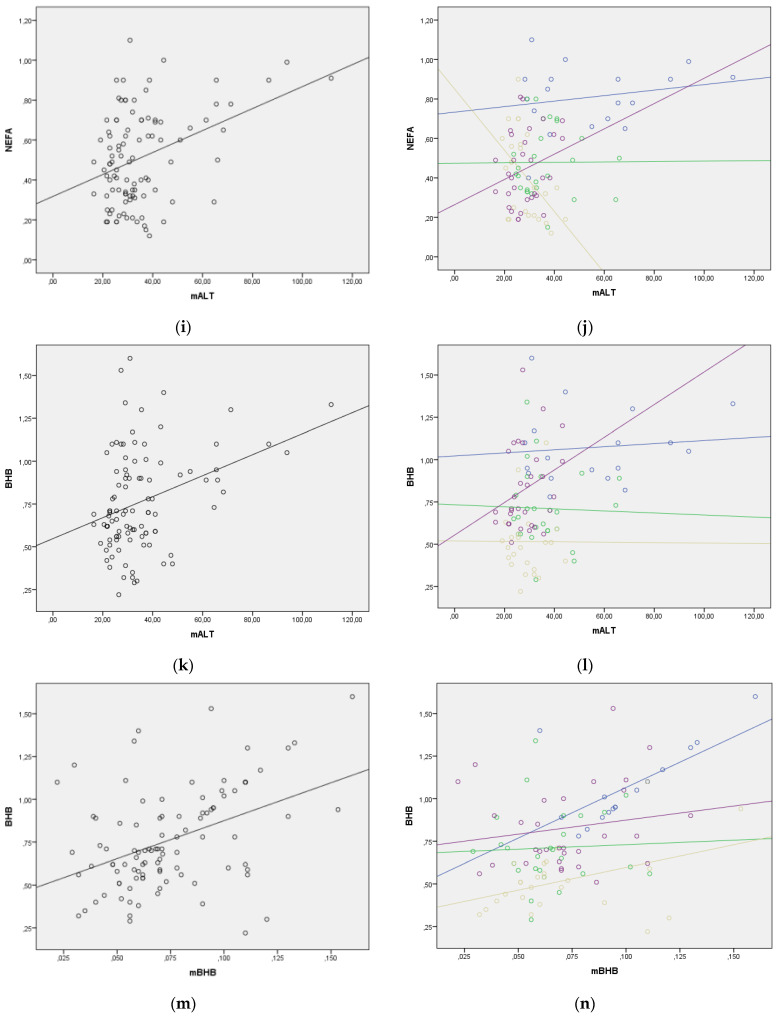
(**a**–**n**) Correlation between blood NEFA and BHB values and milk parameters with lactation period added as a covariate (lactation period and the color of both the line and the figures: early lactation-Group 1—blue; full lactation-Group 2—green; mid lactation-Group 3—golden; late lactation-Group 4—violet). (**a**) Correlation between milk fat and blood NEFA in whole lactation; (**b**) Correlation between milk fat and blood NEFA with lactation period as covariate; (**c**) Correlation between milk fat and blood BHB in whole lactation; (**d**) Correlation between milk fat and blood BHB with lactation period as covariate; (**e**) Correlation between milk AST (mAST) and blood NEFA in whole lactation; (**f**) Correlation between milk AST (mAST) and blood NEFA with lactation period as covariate; (**g**) Correlation between milk AST (mAST) and blood BHB in whole lactation; (**h**) Correlation between milk AST (mAST) and blood BHB with lactation period as covariate; (**i**) Correlation between milk ALT (mALT) and blood NEFA in whole lactation; (**j**) Correlation between milk ALT (mALT) and blood NEFA with lactation period as covariate; (**k**) Correlation between milk ALT (mALT) and blood BHB in whole lactation; (**l**) Correlation between milk ALT (mALT) and blood BHB with lactation period as covariate; (**m**) Correlation between milk BHB (mBHB) and blood BHB in whole lactation; (**n**) Correlation between milk BHB (mBHB) and blood BHB with lactation period as covariate.

The regression lines for the statistically significant correlations between blood NEFA and BHB levels and selected milk parameters are presented in Figure 2. The regression lines for milk fat to blood BHB, milk fat to blood NEFA, and milk BHB to blood BHB relationships showed good homogeneity with the similar slopes through all four lactation periods. However, in other relationships, the regression lines changed their slope, indicating that lactation period should be taken into consideration when evaluating lipolysis and ketogenesis using milk parameters (Figure 2). The change in the value of NEFA and BHB in the function of enzymes in milk was much greater in the later periods of lactation. This requires additional research.

## 4. Discussion

Modern dairy farming often results in forced milk production, giving rise to metabolic disorders in cows. To predict such disorders and related subclinical diseases, it is necessary to establish the physiological ranges of biochemical parameters in a clinically healthy herd [26,32,33]. 

This study examined the associations between different blood and milk metabolic biomarkers in dairy cows at various stages of lactation by correlation analyses, focusing on the relationship between blood NEFA and BHB levels and milk metabolic parameters using single linear regressions. Blood NEFA as the best indicator of negative energy balance (NEB) and lipomobilization during lactation [12,25,34,35] was significantly elevated (*p* < 0.01) in early lactation cows compared to mid, full and late lactation cows. Blood and milk serum concentrations of BHB, another indicator of energy metabolism in early lactation cows, were also significantly higher (*p* < 0.01) than in the other groups of lactation cows, indicating intense fat reserve mobilization. Subclinical ketosis and clinical ketosis involve blood serum BHB levels above 1.2 mmol/L and above 2.9 mmol/L, respectively [2,11,28,36]. Early lactation cows had indicative BHB values (1.07 ± 0.22 mmol/L), without the presence of clinical signs. Blood and milk concentrations of the lipomobilization and ketogenesis marker, BHB, were positively correlated (*p* < 0.01) in this study, which is in agreement with a previous study [37]. Serum BHB and NEFA levels in puerperal cows clearly indicated the presence of some degree of ketogenesis and hepatic fatty infiltration due to intense lipomobilization in the post-partum period [38,39,40,41,42].

In the present study, glycemia values in mid, full and late lactation cows were within the physiological range of 2.5 to 4.2 mmol/L [32]. Nevertheless, glucose levels decreased in early lactation cows compared to the other lactation groups of cows (*p* < 0.01). This hypoglycemia in early lactation cows previously reported in various studies [7,10,42,43] may be associated with a lower liver gluconeogenesis process and with the sudden activity of the mammary gland and increased lactose synthesis. During puerperium, decreased values (*p* < 0.01) were also found for the other blood biochemical parameters, at least partially synthesized in the liver, such as TG, TChol, albumin, globulin, and urea. This indicated an increased accumulation of TG and TChol in hepatocytes in puerperal cows, most likely due to the depleted synthesis of VLDLs in the liver [44].

In cows with liver cell damage, nitrogen metabolism parameters, including uremia, proteinemia and albuminemia, decreased [8,33,45]. Although the levels of these three parameters in cows during the lactation period in the present study were within the physiological range, i.e., 60–80 g/L for proteinemia, 30–40 g/L for albuminemia and 1.66–6.66 mmol/L for uremia [32], they declined in puerperal cows compared to lactation females, which confirmed that the synthesis of these parameters in the liver was reduced due to the development of fatty infiltration of the liver [8,17,18,25,45]. This statement was confirmed by the significantly higher (*p* < 0.01) blood concentration of TBil in early lactation cows, which experienced a decline in the excretory capacity of the liver during fatty liver development. During the first month of lactation, 5–10% of high-yielding dairy cows suffer from severe hepatic lipidosis, and 30–40% of cows develop mild hepatic lipidosis [8], which indicates that almost 50% of these cows are at risk for metabolic disorders. Fatty infiltration of the liver causes lesions in the hepatic tissue and a general increase in the levels of the enzymes indicating hepatocyte injury, i.e., AST, GGT, and GLDH [6,17,18,46].

In this experiment, the activities of blood and milk serum AST, ALT, ALP and LDH were significantly higher (*p* < 0.01) in early and full lactation cows than in the other two groups of cows, suggesting mild fat infiltration of liver cells and a release of these enzymes in circulation, as induced by lipomobilization. Changes in blood and milk AST, ALT, ALP, LDH and GGT activities at different lactation stages indicated a mild degree of hepatic lesions in early lactation cows, probably due to fat infiltration. There were significant positive correlations between blood and milk serum AST (r = 0.450; *p* < 0.01), ALT (r = 0.649; *p* < 0.01), ALP (r = 0.344; *p* < 0.01) and GGT (r = 0.211; *p* < 0.05) activities in this study (Table 3). These results are supported by the reports of other authors [21,22,23,28,47], who showed that milk enzyme activities can be good indicators of lipid mobilization and ketogenesis in cows during lactation for early detection of subclinical disease. The high correlation coefficient of the work agrees with the finding of Liu et al. [21], and the high significance arises due to the large number of samples examined in this experiment. The activities of some milk enzymes were strongly positively correlated (*p* < 0.05) with negative energy balance (NEB) and lipomobilization biomarkers (NEFA, BHB), as well as with the blood biomarkers of the excretory capacity of the liver (TBil), and negatively correlated (*p* < 0.05) with the parameters of the functional state of the liver (glucose, TChol and TG).

The stage of lactation has a significant effect on raw milk composition in dairy cows [30,33]. Risk factors for developing general or mammary gland diseases should be examined in combination with fat and protein as major milk components by determining milk urea as an indicator of a balanced diet [47]. In this research, milk fat content was lower (*p* < 0.05) in early lactation cows (Group 1) than in the other three groups of lactating cows. Milk protein and lactose levels significantly declined (*p* < 0.01) from early to late lactation. There was no significant difference (*p* > 0.05) in milk urea levels between the experimental groups of cows. Milk fat levels are dependent on ration composition. Early lactation cows tend to mobilize body reserves while ingesting rations that are low in effective fiber; accordingly, milk fat levels decrease [48,49,50]. Milk fat can also be produced from volatile fatty acids and, inter alia, from the acetic acid formed in the rumen of cows [51]. Low milk fat levels are induced by a lack of the major precursor, acetic acid, in rumen [26,33,52]. Lactation performance and milk fat synthesis increased with branched-chain volatile fatty acid supplementation by improving ruminal fermentation, nutrient digestibility and mRNA expressions of genes related to milk fat synthesis [52].

It is well known that milk composition of dairy cows is affected by their energy balance (EB), especially during early lactation [42]. Cows in NEB mobilize their adipose tissue, which elevates blood NEFA levels [9,41]. This increase in NEFA supply for milk fat synthesis causes increases in milk fat content and milk fat:protein ratio (FPR) during the lactation period. Moreover, lipomobilization leads to changes in milk fatty acids composition [29,42,53]. The composition of milk fatty acids and its changes are promising EB predictors because they can be measured from routinely collected test-day milk samples [29,53,54]. Milk fatty acids are related to ruminal pH and subacute ruminal acidosis [55]. The authors found that milk fatty acids, alone, are better EB predictors than milk yield, milk FPR, and body traits combined.

In this study, milk fat content during lactation was positively correlated (*p* < 0.05) with total protein, glucose and TG levels, and negatively correlated with TBil, ALT, LDH, ALP, BHB and NEFA in the blood serum. These results were also confirmed by the regression lines for the relationships milk fat: blood BHB, milk fat: blood NEFA, milk BHB: blood BHB, which showed good homogeneity with similar slopes through the four stages of lactation. These correlation and regression relationships clearly indicate that milk fat was directly correlated with blood glucose, protein and triglycerides over the entire course of lactation, and negatively correlated with the lipomobilization and NEB parameters (NEFA, BHB), i.e., indicators of the functional and morphological state of the liver (enzymes, bilirubin). These results are consistent with studies showing that milk fat content in dairy cows during the first months of lactation can be monitored with moderately high accuracy using routine milk measurements [26,29,42,53,54]. Low milk fat content is commonly used in farms to indicate subacute ruminal acidosis and predict the effectiveness of diet structure for chewing [48,49,50]. Milk fat can be a very interesting indicator for assessing metabolic stress in the form of lipolysis and ketogenesis, because it correlates with NEFA and BHB in the blood, so that the slope of the regression curve is similar in all periods of lactation, which gives additional value to this parameter. In earlier research, it was found that in cows with metabolic disease in early lactation there is a change in the value of milk fat and the milk fat to protein ratio [27]. Milk fat is an indicator to which special attention must be paid in the future, especially because this parameter is measured routinely and daily. Energy balance of cows could be predicted by milk traits obtained by herd testing, and milk BHB concentration and blood NEFA predictions are potentially useful tools for management purposes [56,57].

An increase in milk protein content and a decrease in milk fat content cause subclinical acidosis [58], as shown in this experiment in early lactation cows. On the other hand, considering the high sensitivity of the fat:protein ratio (FPR) > 1.42 or lower (>1.35 or >1.25), these cut-offs could be used as a screening test to avoid testing all the cows if a herd is systematically monitored for subclinical ketosis (SCK) [59]. Milk urea and protein levels are indicators of metabolic nitrogen balance, which characterizes the health and reproductive ability of cows [60]. The negative regression dependency reported for the milk protein to milk urea ratio was stronger in early lactation but decreased in mid and late lactation [61]. In this study, there was a significantly positive correlation (*p* < 0.05) between milk protein and blood GGT activity. The energy to protein ratio is the most important nutritional factor in cow rations. Urea level increases with increasing intake of rumen degradable protein, but also when energy in rations is lacking, since no optimal amount of protein can be utilized from the ration due to decreased activity of rumen bacteria. As the feed energy supply increases, the concentration of urea in milk decreases [26,62]. In early lactation (Group 1), milk urea levels were non-significantly lower (*p* > 0.05) than in the other three lactation periods due to the decreased energy supply (NEB) through diet in the puerperal period, as well as due to increased lipomobilization from body reserves. Blood urea tended to have a significant positive relationship with milk fat and a significant negative relationship with milk protein, and a mainly positive relationship with milk lactose [63]. Milk lactose content was significantly lower (*p* < 0.01) in early and late lactations than in full and mid lactation. The confirmed decrease in milk lactose content and yield during lactation is in agreement with the results of Henao-Velásquez et al. [64] who explained this relationship as a decrease in milk yield in continuous lactation when milk secretion is regulated by lactose synthesis in the mammary gland.

This study emphasizes that milk is the most promising matrix for the purpose of diagnosing subclinical metabolic disease because it is easy to sample and allows whole herd testing during routine recording. The latest results, published several weeks ago, confirm the possibility of predicting blood metabolic parameters from milk samples obtained during routine milking [65]. The author found moderate correlations between the observed and predicted parameters, with which our result agrees. New research should further investigate metabolic stress using milk samples as a function of lactation stage to assess the health and productivity characteristics of cows.

## 5. Conclusions

Dynamic changes in metabolite values in blood and milk during lactation are identical, which confirms the possibility of using metabolic parameters from milk for the purposes of assessing metabolic status in cows. Correlation, regression and covariance analysis between blood and milk metabolic parameters confirms that milk parameters can be indicators in the evaluation of the metabolic status of cows. Parameters from milk are a significant indicator of the metabolic stress of cows because they correlate with the parameters of lipolysis and ketogenesis in the blood of cows. It is necessary to know the lactation period in order to correctly interpret the mutual relationships of metabolic parameters. Milk samples are obtained non-invasively, which additionally makes it suitable for evaluating the metabolic status in everyday practice, when the milk comes from a healthy udder.

## Figures and Tables

**Table 1:** Blood metabolic parameter in early (Group 1), mid (Group 2), full (Group 3) and late (Group 4) lactation dairy cows.

Blood Parameters	Lactation Period				ANOVA	LSD
	Early (1)	Mid (2)	Full (3)	Late (4)		
Glucose (mmol/L)	1.91 ± 0.73	2.34 ± 0.44	2.58 ± 0.73	2.74 ± 0.74	*p* < 0.01	1:2,1:3,1:4, 2:4
NEFA (mmol/L)	0.81 ± 0.16	0.48 ± 0.17	0.41 ± 0.22	0.44 ± 0.18	*p* < 0.001	1:2,1:3,1:4
TG (mmol/L)	0.11 ± 0.06	0.17 ± 0.06	0.18 ± 0.06	0.19 ± 0.08	*p* < 0.001	1:2,1:3,1:4
TChol (mmol/L)	3.03 ± 0.91	4.14 ± 1.12	4.02 ± 1.56	4.02 ± 1.56	*p* < 0.01	1:2,1:3,1:4
BHB (mmol/L)	1.07 ± 0.22	0.71 ± 0.23	0.52 ± 0.19	0.82 ± 0.25	*p* < 0.001	1:2,1:3,1:4, 2:3, 3:4
TP (g/L)	63.1 ± 6.8	68.2 ± 6.2	73.94 ± 6.53	68.9 ± 5.54	*p* < 0.001	1:2,1:3,1:4, 2:3,3:4
Albumin (g/L)	28.8 ± 5.11	31.9 ± 5.1	31.8 ± 6.4	31.6 ± 4.34	NS	/
Globulin (g/L)	34.3 ± 7.6	36.3 ± 6.4	42.1 ± 8.7	37.4 ± 6.4	*p* < 0.01	1:3,2:3,3:4
Urea (mmol/L)	3.23 ± 0.91	4.50 ± 1.39	4.56 ± 0.91	4.33 ± 1.45	*p* < 0.01	1:2,1:3,1:4
TBil (µmol/L)	20.42 ± 11.24	6.41 ± 5.03	5.84 ± 4.39	7.06 ± 2.61	*p* < 0.001	1:2,1:3,1:4
AST (IU/L)	134.8 ± 37.3	100.90 ± 30.1	99.00 ± 37	95.20 ± 31.7	*p* < 0.001	1:2,1:3,1:4
ALT (IU/L)	59.16 ± 19.87	36.00 ± 9.46	28.60 ± 5.63	28.64 ± 6.85	*p* < 0.001	1:2,1:3,1:4,2:3,2:4
LDH (IU/L)	1795 ± 942	1647 ± 329	1389 ± 224	1312 ± 285	*p* < 0.001	1:3,1:4, 2:4
ALP (IU/L)	108.17 ± 27.9	85.88 ± 16.7	75.84 ± 15.8	71.00 ± 18.3	*p* < 0.001	1:2,1:3,1:4
GGT (IU/L)	24.61 ± 6.1	21.19 ± 6.1	23.24 ± 10.8	27.19 ± 6.6	*p* < 0.05	2:4

NS—non-significant, *p* > 0.05; NEFA—non-esterified fatty acids; BHB—β-hydroxybutyrate; TG—triglycerides; TChol—total cholesterol; TP—total protein; TBil—total bilirubin; AST—aspartate aminotransferase; ALT—alanine aminotransferase; ALP—alkaline phosphatase; GGT—gamma-glutamyl transferase; LDH—lactate dexydrogenase.

**Table 2:** Milk metabolic parameters in early (Group 1), mid (Group 2), full (Group 3) and late (Group 4) lactation dairy cows.

Milk Parameters	Lactation Period				ANOVA	LSD
	Early (1)	Mid (2)	Full (3)	Late (4)		
Fat (%)	3.04 ± 0.59	3.35 ± 0.61	3.87 ± 0.53	4.16 ± 0.59	*p* < 0.001	1:3,1:4,2:3,2:4,3:4
Protein (%)	3.24 ± 0.50	2.94 ± 0.23	3.09 ± 0.22	3.40 ± 0.41	*p* < 0.001	1:2,2:4,3:4
Lactose (%)	4.75 ± 0.09	4.92 ± 0.1	4.82 ± 0.1	4.69 ± 0.11	*p* < 0.001	1:2,2:4,3:4
Urea (mg/dL)	10.84 ± 6.88	14.24 ± 5.96	12.72 ± 4.31	14.2 ± 6.86	NS	/
AST (IJ/L)	125.7 ± 71.7	62.3 ± 35.1	59.1 ± 32.4	69.6 ± 58.1	*p* < 0.001	1:2,1:3,1:4
ALT (IJ/L)	54.8 ± 24.9	36.3 ± 11.2	28.3 ± 6.9	28.4 ± 7.03	*p* < 0.001	1:2,1:3,1:4,2:3,2:4
ALP (IJ/L)	947.1 ± 543.3	629.5 ± 338.5	568.2 ± 325.8	670.8 ± 358.7	*p* < 0.05	1:2,1:3,1:4,2:4,3:4
GGT (IJ/L)	561.9 ± 217.1	446.9 ± 161.7	571.3 ± 325.8	670.8 ± 135.9	*p* < 0.01	2:3,2:4
LDH (IJ/L)	316.4 ± 156.4	205.6 ± 132.6	200.6 ± 123.2	213.6 ± 117.1	*p* < 0.05	1:2,1:3,1:4
BHB (mmol/L)	0.10 ± 0.02	0.06 ± 0.02	0.09 ± 0.01	0.07 ± 0.02	NS	/

NS—non-significant, *p* > 0.05; NEFA—non-esterified fatty acids; BHB—β-hydroxybutyrate; TG—triglycerides; TChol—total cholesterol; TP—total protein; TBil—total bilirubin; AST—aspartate aminotransferase; ALT—alanine aminotransferase; ALP—alkaline phosphatase; GGT—gamma-glutamyl transferase; LDH—lactate dexydrogenase.

**Table 3:** Correlation between milk composition and diagnostic blood metabolic parameters.

Correlation	Milk Fat	Milk Protein	Milk Lactose	Milk AST	Milk ALT	Milk ALP	Milk GGT	Milk LDH	Milk BHB	Milk Urea
**Blood TP**	0.201 *	−0.039	−0.023	−0.194	−0.191	−0.022	0.007	0.016	0.019	−0.04
**BloodAlbumin**	0.322 **	0.064	−0.071	−0.236*	−0.144	0.063	0.109	−0.003	0.027	−0.029
**BloodGlobulin**	−0.037	−0.081	0.028	−0.016	−0.078	−0.064	−0.069	0.017	−0.001	−0.016
**Blood TBil**	−0.273 **	−0.068	0.014	0.291 **	0.299 **	0.141	−0.023	0.105	0.136	−0.048
**Blood AST**	−0.173	−0.094	0.052	0.450 **	0.185	0.035	−0.115	0.029	0.043	−0.135
**Blood ALT**	−0.323 **	−0.011	0.025	0.266 **	0.649 **	0.262**	−0.004	0.161	0.065	−0.089
**Blood LDH**	−0.338 **	−0.133	0.171	0.06	0.347 **	0.073	−0.135	0.116	0.022	0.011
**Blood ALP**	−0.282 **	−0.08	−0.049	0.399 **	0.492 **	0.343**	0.01	0.338 **	0.039	−0.322 **
**Blood GGT**	0.12	0.212 *	−0.143	0.036	0.107	0.163	0.211 *	−0.017	0.112	−0.147
**Blood Glucose**	0.393 **	0.173	−0.076	−0.214 *	−0.256 *	−0.087	0.163	−0.118	0.047	0.145
**Blood Urea**	0.114	−0.005	−0.038	−0.242 *	−0.166	0.03	0.07	0.002	−0.099	−0.076
**Blood TChol**	0.159	0.041	−0.034	−0.291 **	−0.137	−0.137	−0.056	−0.065	0.054	0.106
**Blood TG**	0.304 **	−0.055	−0.014	−0.208 *	−0.325 **	−0.004	−0.036	−0.05	−0.078	0.004
**Blood BHB**	−0.228 *	0.088	−0.085	0.308 **	0.342 **	0.177	−0.036	0.133	0.425 **	−0.087
**Blood NEFA**	−0.324 **	−0.007	0.194	0.269 **	0.381 **	0.084	−0.098	0.097	0.181	−0.005

*—statistically significant correlation (*p* < 0.05); **—highly significant correlation (*p* < 0.01).

## Data Availability

The data presented in this study are available in the article.
